# Applying the Modified Ten-Group Robson Classification in a Spanish Tertiary Hospital

**DOI:** 10.3390/jcm13010252

**Published:** 2023-12-31

**Authors:** Serena Gutiérrez-Martínez, María Nélida Fernández-Martínez, José Manuel Adánez-García, Camino Fernández-Fernández, Beatriz Pérez-Prieto, Ana García-Gallego, Juan Gómez-Salgado, María Medina-Díaz, Daniel Fernández-García

**Affiliations:** 1 Department of Gynecology and Obstetrics, University Hospital of León, 24071 León, Spain; sgutierrezma@saludcastillayleon.es (S.G.-M.); cfdezfdez@saludcastillayleon.es (C.F.-F.);; 2 Department of Biomedical Sciences, Institute of Biomedicine (IBIOMED), Faculty of Veterinary, University of León, 24071 León, Spain; 3 Department of Gynecology and Obstetrics, University Hospital of Oviedo, 33011 Oviedo, Spain; garciaadajose@uniovi.es (J.M.A.-G.); maria.medina@sespa.es (M.M.-D.); 4 Department of Statistics and Operations Research, University of Leon, 24071 León, Spain; 5 Department of Sociology, Social Work and Public Health, Faculty of Labour Sciences, University of Huelva, 21071 Huelva, Spain; 6 Safety and Health Postgraduate Program, Universidad Espíritu Santo, Guayaquil 092301, Ecuador; 7 Health Research Nursing Group (GREIS), Department of Nursing and Physioterapy, University of León, 24071 León, Spain

**Keywords:** gynaecology, delivery, Robson classification, caesarean section, vaginal surgery

## Abstract

Background: Caesarean section is necessary to save the lives of mothers and newborns at times, but it is important to perform it only when it is essential due to all the risks involved. This study aimed to examine the rate of caesarean sections performed at a tertiary hospital using the Robson classification to detect methods for the detection of and/or reduction in these caesarean section rates. Methods: A descriptive, cross-sectional study of a retrospective database was carried out. Results: A total of 10,317 births were assessed. The Robson classification was used to assess these interventions and verify whether the indication for performed caesarean sections was appropriate. In total, 2036 births by caesarean section were performed in the whole sample. The annual caesarean section rate varied between 18.67% and 21.18%. Conclusions: Caesarean sections increased by about 20% in 2021 compared to 2020 even though the trend over the years of study was decreasing. Vaginal delivery after caesarean section is a reasonable and safe option. Caesarean section rates could be improved, mostly in Robson’s Group 2. The Robson classification facilitated progress in the implementation of measures aimed at improving care and adjusting caesarean section rates.

## 1. Introduction

Caesarean section is a surgical intervention, which, like any surgery, is accompanied by non-negligible risks that must be considered, including increased maternal mortality rates of 5.6/100,000 compared to 1.6/100,000 for a vaginal delivery [1].

Caesarean delivery is a surgical intervention that can be vital and life-saving in specific situations during pregnancy and childbirth [2], although it is associated with possible complications and adverse outcomes such as bleeding, anaemia, prolonged hospitalisation, infection or dehiscence of the surgical wound, urinary tract infections, and endometritis, even resulting in maternal death due to haemorrhages or complications of pre-existing pathologies [3].

Post-caesarean paralytic ileus is a rare complication, but it can occur as a result of surgery. Paralytic ileus refers to a decrease in intestinal motility that can cause the obstruction and retention of contents in the gastrointestinal tract. Although not exclusive to caesarean sections, it can occur after a caesarean section due to manipulation of the bowel during surgery and it may be associated with increased maternal morbidity in the short term [4]. In the case of repeated caesarean sections, the risk of complications is higher: mainly placental accretism (abnormal adhesion of the placenta to the endometrium that complicates surgery and increases the possibility of postpartum haemorrhage), and intraoperative complications such as intestinal or bladder lesions [5]. It is true that there is no universally established maximum number of C-sections a woman can have before the risks of serious complications increase significantly. The uterus’ ability to heal and withstand the stress of multiple C-sections varies from person to person, and individual and medical factors play a role [6].

Caesarean section also has an increased risk of certain complications in newborns compared to vaginal delivery such as respiratory distress, higher rates of neonatal intensive care admissions, prolonged hospitalisations, and a lower Apgar score [7]. The adaptation to the extrauterine life of the newborn is different in caesarean section compared to vaginal delivery, since the caesarean section abruptly interrupts this natural process, so that the stimulus to initiate breathing is not the passage through the birth canal but tactile stimulation or positive pressure ventilation [8]. Some meta-analyses even state that caesarean sections significantly increase the risk of respiratory infections and asthma in the newborn [9].

Caesarean sections may also lead to possible complications in future pregnancies, such as premature births, uterine rupture, placental implantation abnormalities, or excessive maternal bleeding, which may require a hysterectomy [10]. Thus, they often cause significant and potentially permanent complications and disability, or even death, especially in settings where facilities or capacity to perform surgery safely are lacking. Ideally, caesarean sections should be performed only when medically necessary [11].

Worldwide, the rate of CS is estimated to be about 21%. In sub-Saharan Africa, an average of 5% is estimated, indicating underuse, but the average of 42.8% in Latin America and the Caribbean is suggestive of overuse. Monitoring within-country variation is also crucial and policy-makers should consider the use of monitoring strategies and systems, such as Robson classification, to evaluate trends of CS rates and maternal and infant outcomes in a more action-oriented and meaningful manner [12]. In 1985, the World Health Organization (WHO) stated that there was no justification for any region of the world to have a caesarean section rate above 10–15% [13]. However, current WHO recommendations advise that every effort should be made to perform a caesarean section whenever necessary, rather than focusing on trying to achieve a certain rate [11]. One of the objectives promoted by the WHO for the year 2030 is to reduce maternal and infant morbidity and mortality, and one of the suggested ways to achieve this is by avoiding clinically unnecessary caesarean sections [14]. A study that analysed the caesarean section rates of the 194 WHO member states established 19% of livebirths by caesarean section as a rate associated with lower maternal or neonatal morbidity and mortality, meaning that the previous rate of 15% of caesarean sections set by the WHO in 1985 would be too low [15]. The National Institute of Statistics published a perinatal mortality rate in Castilla y León of 4.78 deaths per 1000 live births in 2022 [16].

According to the National Statistics Institute (INE, for its acronym in Spanish), in 2019, there were a total of 360,617 births in Spain, of which 88,804 were by caesarean section, representing a caesarean section rate of 25% of births [17]. The rates differ between communities and municipalities: in the Leon Hospital, the caesarean section rate in 2019 was 18.76%, from data collected directly from the computer application of the hospital’s birth recording system; in 2020, this rate remained at 18.82%, and, in 2021, it increased to 21.08%.

The rate of caesarean sections in Spain in private centres was 35% compared to 23% in public centres in 2015 [14]. A systematic review of 18 articles showed a higher risk of caesarean section in private hospitals than in public hospitals [18].

The WHO suggests using the Robson classification system as a global standard for assessing and comparing caesarean section rates and for monitoring them within healthcare facilities over time, and between facilities [11]. This classification allows each delivery to be categorised into one of 10 different groups [19]. Table 1 shows the characteristics of each group according to the data collected: singleton or multiple pregnancy, parity (nullipara or multipara), onset of labour (spontaneous, induced, or pre-labour caesarean section (CS)), gestational age (preterm or term), history of previous caesarean section, type of foetal lie and presentation (cephalic, breech, or transverse or oblique lie), and whether the caesarean section was performed as planned (before the onset of labour) or during the course of labour.

The ten groups into which the population served is divided are mutually exclusive and totally comprehensive, so that each woman can be classified into one, and only one, of the 10 groups and no woman will be left out of the classification. This Robson classification is now used in more than 50 countries [17] and is supported by both the WHO [20] and the International Federation of Gynaecology and Obstetrics (FIGO) [21].

The present study has two objectives: firstly, to categorise the caesarean sections performed at Leon Hospital between 1 January 2016 and 31 December 2021 according to Robson’s classification and to study how the composition of each group has evolved; secondly, to analyse the caesarean section rate throughout the study period, with a special emphasis on the possible detection of measures for improving caesarean rates and complications.

## 2. Materials and Methods

### 2.1. Design

A descriptive, cross-sectional study of a retrospective database was carried out. The total number of caesarean sections performed at the Leon Hospital between 1 January 2016 and 31 December 2021 was assessed.

### 2.2. Scope and Study Population

The Leon Hospital is a tertiary hospital, a reference centre for obstetrics and gynaecology in the province of Leon, to which pregnant women of less than 32 weeks of gestation from all over the province and all women with pathologies susceptible to prenatal and/or neonatal treatment are referred. During the studied 6-year period, a total of 10,395 deliveries were registered, of which 78 were rejected and excluded from the study due to errors in data collection. Thus, a total of 10,317 births were assessed.

### 2.3. Procedure

Data were collected on the number of births and caesarean sections in each year, the number of women in each Robson group, the number of caesarean sections in each group, and the caesarean section rate by group. The absolute and relative contribution to the total number of caesarean sections was examined, describing which group contributed most and the variation in births and caesarean sections across years. The absolute contribution referred to the number of caesarean sections in each group with respect to the total number of women delivered in the hospital, while the relative contribution referred to the number of caesarean sections in each group with respect to the total number of caesarean sections in the hospital.

The data were collected from the Hospital de León’s birthing app, a standardised database that midwives fill in personally after each birth. The application systematically collects data on filiation, parity, personal and obstetric history of interest, information related to the current pregnancy, such as the date of last period, whether it is a single or multiple pregnancy, and data related to the birth (date, type of analgesia used, presence of episiotomy, complete perineum tear, and complications arising during birth), and data of the newborn (its gestational age, sex, weight, Apgar, and umbilical cord pH).

The protocols of the Hospital of León include the following as reasons for the indication of a caesarean section: stationary delivery diagnosed after 4 h with the same vaginal examination in an active pregnant woman with a ruptured amniotic sac and sufficient uterine dynamics of 4–5 contractions every 10 min under the effect of oxytocin; induction failure diagnosed as no initiation of active labour with at least 4 cm dilation and erased cervix after 12 h of amniotomy and administration of oxytocin; anomalous breech or transverse presentation; risk of loss of foetal or miscellaneous well-being reserved for pregnant women who refuse a vaginal birth after a previous caesarean section despite being recommended by the medical team.

The obstetrics and gynaecology team at the Hospital of León works to reduce the rate of caesarean sections through weekly audits and reviews of the correct application of protocols and indications.

### 2.4. Ethical Aspects

The data were collected from the birth data application of the hospital’s obstetrics and gynaecology department. Data were previously anonymised and collected by authorised personnel. The protocol complied with the Declaration of Helsinki and has been approved by the Ethics Committee for Research with Medicines of the Health Areas of Léon and Bierzo (Spain) with the number/reference 2105.

### 2.5. Statistical Analysis

Statistical analysis of the data was carried out using a Microsoft Excel spreadsheet and SPSS version 26 (IBM, Armonk, NY, USA). A descriptive analysis of the data was carried out using means and standard deviation. The variables were also presented as percentages. Chi-squared was used as the contrast statistic. A *p* < 0.05 was considered statistically significant.

## 3. Results

During the 2016–2021 period, there were 10,395 deliveries in the hospital, of which 10,317 were considered valid for study. Of these, 2036 were by caesarean section. The mean age of women during this period was 33.1 years.

The annual caesarean section rate varied between 18.67% and 21.18% in the studied period, with a mean caesarean section rate of 19.73%, as can be seen in Table 2.

Table 3 shows data disaggregated by Robson group, including the number of women in each group, the number of caesarean sections, and the caesarean section rate for each group, as well as their contribution, both absolute and relative to the overall caesarean section rate.

The changes in the number of women in the period 2016–2021 compared to 2016 according to Robson groups are shown in Supplementary Table S1. The largest Robson group was group 1 (3008 women), followed by group 3 (2763 women). The third largest group was group 2 (1752 women). It can be seen that the number of women in this group has increased over the years, i.e., more planned caesarean sections and inductions in nulliparous women were performed at Leon Hospital each year. The three groups together account for a total of 7523 deliveries, which represents 72.92% of all analysed deliveries. The least numerous group is group 9, in which 100% of caesarean sections were performed.

In relation to the caesarean section rate by group, the highest rate was found in group 9, where 100% of the cases were caesarean sections, followed by groups 6 and 7, with 98.74% and 98.21%, respectively. Only five breech deliveries were assisted at Leon Hospital in 6 years, mainly due to precipitous or very advanced labour upon admission to the emergency department. Groups 8 and 5 also presented high rates of caesarean sections, with rates of 52.16% and 44.39%, respectively.

The group that contributed most to the overall number of caesarean sections performed at Leon Hospital was group 2, with 28.14% of the total number of caesarean sections, followed by group 5 with 18.27%, and group 1 with 14.64%.

**Figure 1:**
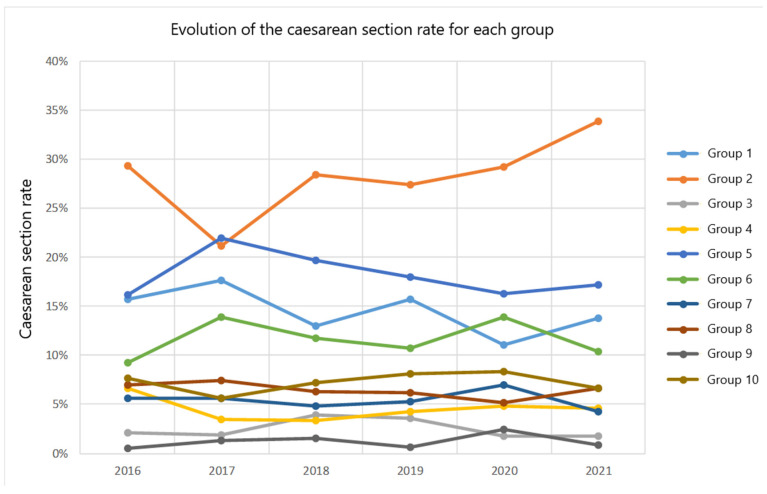
Evolution of the caesarean section rate for each Robson group for the 2016–2021 period.

These data and those of the other groups are shown in Figure 1, which illustrates the evolution of the different groups throughout the study period.

The number of women giving birth at Leon Hospital has been decreasing year on year (taking into account that the variation felt in 2018 compared to 2017 was very small, which indicates that the figures remain practically the same), except in the year 2021, when there was an increase of almost 8%. To assess this increase, Table 4 shows the variations for each group with respect to the previous year, with group 8 standing out with an increase of more than 60% in 2021 with respect to 2020.

As for caesarean sections, a similar behaviour can be observed, with the number of caesarean sections decreasing year on year, but increasing by more than 20% in 2021 compared to 2020.

Concisely, Group 2 is identified as the main contributor to overall caesarean section rates. In addition, the rate of caesarean section in this group exhibits an increasing trend over time, with Group 5 being the second largest contributor to the total number of caesarean sections in the study hospital.

## 4. Discussion

Caesarean sections are effective in saving the lives of mothers and newborns only when medically necessary [11]. The effects of caesarean section rates on other outcomes such as stillbirths, maternal and perinatal morbidity, paediatric outcomes, and psychological or social well-being are unclear. Further research is needed to understand the health effects of caesarean sections on some immediate and future outcomes [11].

Several studies have found that the main reason for performing a caesarean section is a previous caesarean section [22]. The American College of Obstetricians and Gynecologists (ACOG) issued a practice bulletin in 2019 that evaluated the risks and benefits of attempting vaginal birth after caesarean section and provided practical guidelines for counselling and treating patients who wished to opt for attempted vaginal birth after caesarean section. In this paper, it was established that vaginal birth after caesarean section is associated with lower rates of bleeding, thromboembolism, and infection, as well as a shorter recovery period compared to women who choose elective repeat caesarean section. Thus, it was highlighted that vaginal birth after caesarean section may reduce the risk of maternal consequences following multiple caesarean sections [9].

Although vaginal delivery following caesarean section has been argued to be a safe option [23,24], the number of women opting for this procedure after a caesarean section has decreased in recent years due to a fear of uterine rupture [25,26]. At Leon Hospital, the sample of women in Robson’s group 5, which includes pregnant women with previous caesarean sections, has remained more or less stable over the years.

This study presents data on the total of 10,317 births at Leon Hospital between 2016 and 2021, the number of women and caesarean sections in each of the Robson Classification groups, the rate of caesarean sections by group, and the absolute and relative contribution to the overall number of caesarean sections in the hospital during the studied period. It also reports the variation in the number of deliveries and caesarean sections within each year.

A progressive decrease in the number of women who delivered in the hospital can be observed throughout all years. The exception is 2021, when there was a 7.91% increase. The caesarean section rate in 2021 increased by 20.83% compared to 2020. A possible explanation could be the increase in the sample size of Robson’s group 2, since, in these last years, the criteria for induction have been modified (i.e., maternal age of 40 years), thereby increasing the rate of induction procedures at the hospital. Historically, it was believed that induced labour was associated with increased rates of caesarean sections, although this has not been supported by the latest scientific evidence, which does not link induced labour in women over 35 years of age with higher rates of surgical delivery or adverse maternal or neonatal outcomes [27,28]. Others claim that induced labour in pregnant women under 35 years of age is not associated with an increased risk of caesarean section either [29].

When comparing the data from Leon Hospital with those published by other international studies, it was observed that the overall caesarean section rate was lower than the rates published in Malaysian tertiary hospitals, at 23.2% [30]; Australia, 23.5% [25]; Ethiopia, 34.7% [31]; Flanders, 20.9% [32]; Portugal, 25% [33]; Canada, 29.1% [34]; India, 25.5% [35]; Dubai, 33% [36]; and Brazil, 56% [37]. It has also been observed that this rate was higher than the rates reported in other countries such as Norway (16.5%), Sweden (17.1%), Finland (16.2%), and Iceland (15.3%) [38], and similar to published data from Ireland (19.3%) [39].

The distribution of caesarean sections by group was analysed at Leon Hospital and the Spanish regional hospitals of La Ribera, Basque Country, Almeria, and Alicante (studied in the second period).

Firstly, there are differences in the size of Robson’s groups between the Leon setting and the other hospitals being compared. Differences can be observed in the percentage of women out of the overall number of women delivered in each hospital. From the data, it can be deduced that at Leon Hospital, there was a lower proportion of women with spontaneous delivery (groups 1 and 3) and more women with induced delivery or planned caesarean section (groups 2 and 4) than at the hospitals of La Ribera and Alicante [40,41]. In comparison with the Basque Country Hospital, the latter did have a higher proportion of women in group 1, but the difference was not significant in group 2; i.e., in the Basque Country Hospital there were significantly more term deliveries in nulliparous women with spontaneous onset, but no more inductions or planned caesarean sections [42].

In all hospitals except Almeria hospital [41], the size of Robson’s group 5 was significantly smaller than that of Leon Hospital, which means that there was a higher proportion of multiparous women with a previous caesarean section in that hospital.

In terms of the mean total percentage of caesarean sections, both the Basque Country Hospital, with 13.30%, and Almeria Hospital, with 16.14%, had significantly lower percentages of caesarean sections than Leon Hospital, with 19.73%. In the second study period, Alicante Hospital, with 20.92%, also had a lower percentage of caesarean sections than Leon Hospital.

In Leon Hospital, the Robson groups with the highest percentages of caesarean sections were groups 6 (98.74%), 7 (98.21%), and 9 (100%), similar to in the rest of the hospitals being compared, i.e., Hospital de La Ribera (95% caesarean sections in group 6, 86.7% caesarean sections in group 7, and 100% caesarean sections in group 9) [40], Hospital de Alicante (96.8% caesarean sections in group 6, 88.4% caesarean sections in group 7, and 100% caesarean sections in group 9) [41], Hospital del País Vasco (70.7% caesarean sections in group 6, 73.9% caesarean sections in group 7, and 100% caesarean sections in group 9) [42], and the Hospital de Almería (94.7% caesarean sections in group 6, 98.2% caesarean sections in group 7, and 100% caesarean sections in group 9) [43].

The distribution of caesarean sections by Robson groups showed that La Ribera Hospital had a lower percentage of caesarean sections than Leon Hospital in Robson groups 5, 6, and 7, and a higher percentage of caesarean sections than Leon Hospital in groups 1, 2, 3, and 4 [40].

Across all Robson groups in the Basque Country Hospital, fewer caesarean sections were performed than in Leon Hospital; this percentage was lower in groups 1, 2, 5, 6, 7, and 10 [42]. In Almeria Hospital, differences were only observed in groups 6 and 10, where the percentage of caesarean sections was lower, as well as in group 8, where the percentage of caesarean sections was higher [43]. In Alicante Hospital, lower percentages of caesarean sections were observed than in Leon Hospital in groups 1, 2, and 7 [41].

The Robson group with the highest absolute percentage of caesarean section rates in Leon Hospital was group 2, as well as in La Ribera, the Basque Country, Almeria, and Alicante hospitals [41]. However, the absolute percentage in the Basque Country and Almeria Hospitals was lower than the one identified in Leon Hospital, and no differences were observed with respect to the two remaining settings.

While in Nordic countries, where caesarean section rates are lower than those reported by Leon Hospital, the groups with the highest relative contribution to the overall number of caesarean sections were groups 1, 3, and 4 (together with group 2), in the case of Leon Hospital, they were groups 2 and 5.

According to a systematic review and meta-analysis of the effects of the COVID-19 pandemic on maternal and perinatal outcomes, the rates of spontaneous vaginal delivery, caesarean section, and instrumental delivery did not change significantly [44], although the analysis of this study showed an increase in the number of women in groups 2 and 4.

The main limitation of this study has been the lack of analysis of individual demographic variables of the patients included in the study, as well as some possible risk factors for caesarean section such as maternal age or the presence of concomitant pathology, which may have an influence on the type of onset and termination of labour. On the other hand, a great strength of this study is that it assessed a large sample of deliveries over 6 years, from which all the necessary data to apply the Robson classification were collected in a computerised form.

As future lines of research, it is proposed to analyse whether reducing labour inductions in full-term nulliparous women can improve the caesarean section rate in Robson’s group 2 by maintaining or improving maternal–foetal morbidity and mortality, and to study the indications for caesarean section in Robson’s group 5 (full-term multiparous women with previous uterine scars) to assess modifications in the management of childbirth that may reduce the high rate of caesarean sections in this group.

The main scientific contribution of this study is to show the Robson groups of births at the Hospital of León that are most susceptible to improvement, taking other hospitals with similar characteristics as a reference to serve as a guide for other tertiary level centres. Acquiring a complete understanding of the long-term effects for women, children, and the civilisation itself should be considered a research priority in the next decade [12].

## 5. Conclusions

The Robson classification of caesarean sections is a useful tool for assessing the groups at higher risk of ending up with a caesarean section in the hospital. The Robson classification is a suitable method for assessing the evolution and aetiology of caesarean section rates in order to implement corrective measures to decrease the overall caesarean section rate and achieve better perinatal outcomes.

The Hospital of León presents Robson groups that represent spontaneous births (groups 1 and 3) lower than the hospitals of La Ribera and Alicante, and more induced births (groups 2 and 4 of Robson’s) than those same hospitals.

The annual caesarean section rate at the Hospital of León varied between 18.67% and 21.18% in the studied period, with a mean caesarean section rate of 19.73%. Given that several hospitals in the surrounding area had significantly lower rates, it can be deduced that there is room for improvement.

The objective of this study was to detect and provide improvements in the birthing processes, especially those that are more susceptible to corrected improvement. In this case, special attention should be paid to group 2, as it contributes the most to overall caesarean section rates. Furthermore, the caesarean section rate within this group shows an increasing trend over time. Since group 5 was the second group that contributed the most to the overall number of caesarean sections in the hospital, it may be appropriate to ensure the observance of delivery events in order to avoid prescribing caesarean sections without having complied with all the times described in the protocols.

## Figures and Tables

**Table 1:** Robson 10-group classification system.

1.	Nulliparous women with a single cephalic pregnancy, ≥37 weeks gestation in spontaneous labour.
2.	Nulliparous women with a single cephalic pregnancy, ≥37 weeks gestation who had labour induced or were delivered by CS before labour.
3.	Multiparous women without a previous CS, with a single cephalic pregnancy, ≥37 weeks gestation in spontaneous labour.
4.	Multiparous women without a previous CS, with a single cephalic pregnancy, ≥37 weeks gestation who had labour induced or were delivered by CS before labour.
5.	All multiparous women with at least one previous CS, with a single cephalic pregnancy, ≥37 weeks gestation.
6.	All nulliparous women with a single breech pregnancy.
7.	All multiparous women with a single breech pregnancy including women with previous CS(s).
8.	All women with multiple pregnancies including women with previous CS(s).
9.	All women with a single pregnancy with a transverse or oblique lie, including women with previous CS(s).
10.	All women with a single cephalic pregnancy <37 weeks gestation, including women with previous CS(s).

CS = caesarean section.

**Table 2:** Caesarean section rate for each studied year.

Year	Births	Caesarean Sections	Caesarean Rate
2016	1957	389	19.88%
2017	1775	374	21.07%
2018	1773	331	18.67%
2019	1631	306	18.76%
2020	1530	288	18.82%
2021	1651	348	21.18%
Total 2016–2021	10,317	2036	19.73%

**Table 3:** Robson’s classification of caesarean sections performed at Leon Hospital between 2016 and 2021.

Robson Group	No. of Women in the Group	No. of Caesarean Sections in the Group	Group Caesarean Section Rate (%) *	Absolute Contribution of the Group to Overall Caesarean Section Rate (%) **	Relative Contribution of the Group to Overall Caesarean Section Rate (%) ***
1	3008	298	9.91%	14.64%	2.89%
2	1752	573	32.71%	28.14%	5.55%
3	2763	50	1.81%	2.46%	0.48%
4	770	93	12.08%	4.57%	0.90%
5	838	372	44.39%	18.27%	3.61%
6	239	236	98.74%	11.59%	2.29%
7	112	110	98.21%	5.40%	1.07%
8	255	133	52.16%	6.53%	1.29%
9	24	24	100%	1.18%	0.23%
10	556	147	26.44%	7.22%	1.42%
Total	10,317	2036	19.73%	100%	19.73%

(*) Caesarean section rate over total number of women in the group (caesarean sections/women within each group). (**) Contribution of each group to total deliveries (caesarean sections/total deliveries). (***) Contribution of each group to total caesarean sections (group caesarean sections/total caesarean sections).

**Table 4:** Variation in the number of caesarean sections in the period 2016–2021 compared to the previous year according to Robson groups.

Robson Group	2017	2018	2019	2020	2021
1	8.20%	−34.85%	11.63%	−33.33%	500%
2	−30.70%	18.99%	−10.64%	00%	40.48%
3	−12.5%	85.71%	−15.38%	−54.55%	20%
4	−50%	−15.38%	18.18%	7.69%	14.29%
5	30.16%	−20.73%	−15.38%	−14.55%	27.66%
6	44.44%	−25.00%	−15.38%	21.21%	−10%
7	−4.55%	−23.81%	0.00%	25%	−25%
8	3.70%	−25.00%	−9.52%	−21.05%	53.33%
9	150%	0%	−60%	250%	−57.14%
10	−30%	14.29%	4.17%	−4%	−4.17%
Total	−3.86%	−11.50%	−7.55%	−5.88%	20.83%

## Data Availability

Data are available upon reasonable request to the corresponding author.

## Supplementary Materials

The following supporting information can be downloaded at https://www.mdpi.com/article/10.3390/jcm13010252/s1, Table S1. Changes in the number of women in the period 2016–2021 compared to 2016 according to Robson groups.
