# Supplementary material for: Applying the Modified Ten-Group Robson Classification in a Spanish Tertiary Hospital
Source: J Clin Med. 2023 Dec 31;13(1):252. doi: 10.3390/jcm13010252 (PMC10780088; doi:10.3390/jcm13010252)
Supplement: Supplementary file 1 [file jcm-13-00252-s001.zip › jcm-2744267-supplementary.pdf]

Table S1. Changes in the number of women in the period 2016-2021 compared to 2016 according to Robson groups.

Robson Group	2017	2018	2019	2020	2021
1	-8.77%	-17.70%	-24.24%	-41.31%	-28.23%
2	-18.12%	-4.85%	-23.30%	3.88%	9.39%
3	-12.45%	-9.39%	-13.36%	-33.39%	-32.67%
4	6.73%	25.00%	7.69%	45.19%	55.77%
5	8.57%	1.43%	-0.71%	-15.00%	4.29%
6	43.24%	5.41%	-13.51%	13.51%	-2.70%
7	-13.04%	-26.09%	-21.74%	-13.04%	-39.13%
8	-8.33%	-18.75%	-16.67%	-10.42%	-14.58%
9	150%	150%	0%	250%	50%
10	-29.20%	-21.24%	-15.04%	-20.35%	-22.12%
Total	-9.30%	-9.40%	-16.66%	-21.82%	-15.64%
